# Exploring the Relationship Between Flourishing and the Light Triad of Personality in Honduran Adults

**DOI:** 10.3390/bs15040456

**Published:** 2025-04-02

**Authors:** Miguel Landa-Blanco, Nicolle Espinoza-Rivera, Ivin Caballero-Juárez, Raquel Mejía-Sánchez

**Affiliations:** School of Psychological Sciences, Faculty of Social Sciences, National Autonomous University of Honduras, Tegucigalpa 11101, Honduras; nicolle.espinoza@unah.hn (N.E.-R.); ivin_caballero@unah.hn (I.C.-J.); ramejias@unah.hn (R.M.-S.)

**Keywords:** Flourishing, Light Triad, Kantianism, Humanism, Faith in Humanity

## Abstract

Flourishing refers to a state of optimal well-being and life satisfaction, while the Light Triad traits—Kantianism, Humanism, and Faith in Humanity—represent a prosocial and benevolent approach to interpersonal relationships. This study explores the relationship between these traits and Flourishing among adults in the Honduras Central District. A quantitative, non-experimental, cross-sectional methodology with a relational scope was used in a sample of 715 Honduran adults. Results indicate that Kantianism, Humanism, and Faith in Humanity significantly and positively explain the participants’ levels of Flourishing. Men and women reported similar levels of Flourishing, Faith in Humanity, and Kantianism. However, women scored significantly higher in Humanism. This study highlights the role of the Light Triad traits in predicting Flourishing, emphasizing the need for educational and policy initiatives that foster traits like Kantianism, Humanism, and Faith in Humanity for personal and societal development.

## 1. Introduction

Positive psychology focuses on understanding and promoting positive aspects of human experience, such as subjective well-being, personal strengths, and Flourishing. Unlike traditional approaches in psychology, which primarily address disorders and psychological difficulties, positive psychology aims to study and enhance the positive dimensions of life, optimal functioning, and personal growth (Waters et al., 2022). This model goes beyond the classical reductionist approach based on psychopathology and poor psychosocial functioning (Cortés-Ramos & Landa-Blanco, 2021).

Positive psychology has shown a growing interest in understanding personal strengths and how they contribute to psychological well-being (Akin & Akin, 2015). Flourishing is often described as personal development (Bernal Martínez de Soria & Naval, 2023) and balances feeling good, leading a meaningful life, and functioning effectively. Diener’s model posits that Flourishing combines self-perceived success, self-esteem, meaningful interpersonal relationships, optimism, and life purpose (Diener et al., 2010). Given the individual nature of these variables, the study of Flourishing involves a personal evaluation of an individual’s life conditions. Therefore, it is a term associated with multidimensional subjective well-being (Landa-Blanco & Landa-Blanco, 2016).

The study of Flourishing in positive psychology is based on the idea that human life can extend beyond mere survival and adaptation and that individuals have the potential to achieve high levels of well-being and personal development (Kelly-Hedrick et al., 2022). Research in this field aims to understand the factors and processes contributing to Flourishing and develop interventions and practices that promote and enhance individuals’ well-being and quality of life. Therefore, these measures of subjective well-being can be considered indicators of social progress (Voukelatou et al., 2021).

Despite the development of various studies aimed at explaining the “dark” personality traits (Machiavellianism, Narcissism, Sadism, and Psychopathy) (Jones & Paulhus, 2013; Pechorro et al., 2023), research on the more benevolent and humanistic aspects of the psyche remains limited. This study will focus on Light Triad traits, which is a recently emerging construct in psychology that proposes that individuals genuinely embody an orientation of beneficence and love toward others. It is summarized in the integration of three personality traits that manifest in everyday behavior: Kantianism, which refers to viewing individuals as ends in themselves and avoiding using them as mere means to an end; Humanism, which denotes the value and dignity each person holds by their inherent humanity; and Faith in Humanity, which refers to the tendency to believe in the pure goodness of humans. These traits reflect a genuine orientation of beneficence and love toward others (Kaufman et al., 2019).

Previous studies have shown that the Light Triad correlates positively with various variables, such as self-esteem, compassionate attitudes and empathy, openness to experience, acceptance of others, beliefs in the virtuousness and goodness of humans, and life satisfaction (Kaufman et al., 2019). In this sense, a positive childhood environment plays a crucial role in fostering Flourishing, as Benevolent Childhood Experiences (BCEs) have been found to significantly contribute to the development of prosocial traits, such as Kantianism, Humanism, and Faith in Humanity, which in turn promote well-being and personal growth (Landa-Blanco et al., 2024).

Individuals’ internal structure is composed of negative or dysfunctional aspects as well as positive and healthy ones that promote personal growth (Gerymski & Krok, 2019; Toyama et al., 2020). It is important to note that the traits that make up the structures of both the light and dark personality traits can coexist within an individual. In other words, the presence of one does not eliminate or imply the absence of the other in the personality (Kaufman et al., 2019).

Although both Flourishing and the Light Triad have been studied independently, research is scarce exploring the relationship between these two constructs. Given the points outlined above, this study aimed to analyze the bivariate relationship between Flourishing and the traits of the Light Triad (Humanism, Kantianism, and Faith in Humanity) in a sample of adults residing in the Central District of Honduras. In the second phase, a directional analysis was conducted, where the Light Triad traits were considered a predictor variable for Flourishing, while controlling for respondent’s sex and age. This directional approach, with personality traits as predictors of Flourishing, has been used in previous studies (Anglim et al., 2020; Schotanus-Dijkstra et al., 2016). The above is justified by the argument that personality traits are primary characteristics that are relatively stable and precede the expression of Flourishing. The relevance of using The Light Triad as a theoretical work-frame for this research lies in its specific emphasis on characteristics that contribute to positive behaviors and relationships. Furthermore, this model acknowledges that the qualities associated with Flourishing can be complex and context-dependent, allowing for a more comprehensive understanding of well-being. Additionally, it supports a nuanced perspective on Flourishing by recognizing the importance of individual experiences in its development (Lomas, 2016; Maralov, 2024). Thus, the following hypothesis was stated: Flourishing will be significantly predicted by Humanism, Kantianism and Faith in Humanity.

Research on Flourishing and the traits of the Light Triad is important for several reasons. First, understanding the relationship between these constructs can provide valuable insights into the psychological factors that contribute to well-being and personal development. This can have significant implications for promoting mental health and improving the quality of life for individuals (Sofija et al., 2020). Additionally, focusing on a sample of adults residing in the Central District of Honduras provides relevant information for understanding the relationship between Flourishing and the Light Triad traits within the specific cultural context of this population. Adding empirical evidence from a geographically and culturally diverse sample can contribute to the existing scientific literature.

## 2. Materials and Methods

The sample consisted of 715 participants in the study. The average age was 27.37 (*SD* = 10.85), ranging from 18 to 71 years. Of these, 61.68% (*n* = 441) were women and 38.32% (*n* = 274) were men. Participants were selected using a non-probabilistic, convenience, and snowball sampling method. The instruments were digitized using Google Forms, and the survey was disseminated across various social media platforms and academic spaces. Inclusion criteria were (1) being over 18 years old, (2) residing in the Central District of Honduras, and (3) providing informed consent to participate in the study.

The Flourishing Scale was used as the data collection instrument (Diener et al., 2010). The Spanish version of the Flourishing Scale was used, which consists of eight items, including the following: “People respect me”, “I lead a meaningful and purposeful life”, and “I am competent and capable in activities that are important to me”. Each item was evaluated using a 7-point Likert scale (1 = strongly disagree; 7 = strongly agree) with higher scores indicating greater Flourishing. This instrument has a unidimensional structure. Based on the sample data, the Flourishing Scale demonstrates high internal consistency, ω of McDonald = 0.90, 95% *CI* [0.89; 0.91]. The Spanish version of this instrument has solid psychometric properties in the Honduran population, including factorial validity, convergent validity, divergent validity, internal consistency, test–retest reliability, and structural invariance based on the participants’ sex (Landa-Blanco et al., 2023).

On the other hand, the Light Triad Scale consists of 12 items equally distributed across three subscales: Humanism, Faith in Humanity, and Kantianism (Kaufman et al., 2019). Some of the items included are “I tend to see the best in people”, “I tend to applaud the successes of others”, and “I like to be authentic, even if this may harm my reputation” among others. Each item was responded to using a Likert-type scale with five response options (1 = strongly disagree, 5 = strongly agree). Higher scores indicate a greater prevalence of the trait being measured. Based on the data collected in this study, the overall Light Triad Scale demonstrates adequate internal consistency, ω of McDonald = 0.79, 95% *CI* [0.76; 0.81]. A subscale analysis reveals that despite Faith in Humanity and Humanism having acceptable internal consistencies (ω = 0.74 and ω = 0.76, respectively), the Kantianism subscale has low reliability (ω = 0.55).

Regarding the data analysis, summative scores were calculated for each scale by adding individual items. Descriptive statistics were conducted for each of the variables included in the study. These descriptions included measures of central tendency, variability, and analysis of absolute and relative frequencies. Internal consistency coefficients—using McDonald’s ω—were then calculated for the Flourishing Scale and the Light Triad Scale. An independent sample Student’s *t*-test was used to compare men’s and women’s scores; this was complemented by reporting their corresponding effect size based on Cohen’s *d* classification (Cohen, 1992). A bivariate correlation analysis was performed using Pearson’s *r*. Finally, multiple linear regression was used to identify how Flourishing scores were predicted by Kantianism, Humanism, Faith in Humanity, the respondent’s age, and gender. All hypothesis testing and confidence intervals were conducted at a 95% confidence level. Jasp 0.19.3 and Jamovi 2.6.26 were used as software to assist in the statistical analysis of the data.

The study was approved by the Research Ethics Committee of the Faculty of Social Sciences of the National Autonomous and registered under protocol CEIFCS-2023-01 as part of a broader study of mental health in Honduran adults. All potential participants were presented with an Informed Consent form that outlined the study title, purposes, participation conditions, inclusion criteria, contact details for the principal investigator, and a statement of risks and benefits. Participants did not receive any money or other compensation for completing the questionnaire. Agreeing to the study conditions was necessary to begin filling out the data. No personally identifiable information was requested, so the surveys were managed with complete anonymity.

## 3. Results

The respondents obtained an average score of 45.72 (*SD* = 8.82) on the Flourishing Scale, 14.48 (*SD* = 3.46) on Faith in Humanity, 35.65 (*SD* = 3.78) on Humanism, and 16.3 (*SD* = 2.90) on Kantianism. It was found that Flourishing scores did not significantly differ according to the respondent’s gender (*p* = 0.25; *d* = 0.09). That is, both men and women reported similar levels of this variable. Similarly, Faith in Humanity and Kantianism scores did not vary based on the participant’s gender. However, women (*M* = 36.17; *SD* = 3.50) reported significantly higher scores than men (*M* = 34.81; *SD* = 4.07) in terms of Humanism (*p* < 0.001, *d* = −0.37), see Table 1.

The results indicate that Flourishing is statistically related to all the traits of the Light Triad traits: Faith in Humanity (*r* = 0.48, *p* < 0.001), Humanism (*r* = 0.32, *p* < 0.001), and Kantianism (*r* = 0.33, *p* < 0.001). As expected, all subscales of the Light Triad Scale also correlated positively with each other (*p* < 0.001), see Table 2.

The directional relationship between these variables was analyzed. Flourishing was identified as the outcome variable, while the predictors included Light Triad traits (Faith in Humanity, Humanism, and Kantianism), age, and participant gender. The overall evaluation of the model indicates that it is significant [*F* (5, 710) = 56.83, *p* < 0.001]. Together, these variables explain 28% of the variance in Flourishing scores. The results indicate that collectively, Faith in Humanity (*β* = 0.94), Humanism (*β* = 0.37), and Kantianism (*β* = 0.49) significantly explain Flourishing scores. The age and gender of the participants did not result in significant coefficients (*p* ≥ 0.05); see Table 3.

## 4. Discussion

This study found that Kantianism, Humanism, and Faith in Humanity significantly and positively explain the levels of Flourishing among the surveyed Honduran adults. These results align with the study by Kaufman et al. (2019), in which a positive relationship was found between the Light Triad and self-transcendent experiences. The findings also align with research in which statistically significant and positive correlations were demonstrated between personality traits, resilience, and Flourishing (Yildirim & Belen, 2019).

On the other hand, both men and women report similar levels of Flourishing, Faith in Humanity, and Kantianism. This finding contrasts with previous international studies, which have identified sex differences in Flourishing scores (Callea et al., 2019; Freire et al., 2020; Fuente-Martín, 2022). However, recent data from Honduras further corroborate that the Flourishing Scale has sex-related measure invariance and that men and women report similar scores (Landa-Blanco et al., 2023). This highlights the importance of the current study, evidencing the differences between international literature and contextualized data from Honduras. Furthermore, women scored significantly higher than men in Humanism. This aligns with a study involving over 2000 participants, which showed that women, compared to men, place greater value on social connection, which is a key component of Flourishing (Lee et al., 2021). Recognizing these differences is vital for leveraging women’s strengths, particularly empathy, to promote leadership and create community support networks, ultimately benefiting the community’s well-being.

The results suggest the importance of considering personality’s cognitive, affective, and ethical aspects when examining personal Flourishing. Light Triad traits may be crucial in promoting Flourishing and psychological well-being. The findings support the idea that Flourishing is not limited to the experience of positive emotions but also involves values and attitudes related to ethics and trust in human potential. Additionally, the results highlight the importance of promoting Light Triad traits in society. This implies the implementation of public policies and educational programs that foster trust in human potential, humanism, and an ethical view of interpersonal relationships. These initiatives could create a more favorable social environment for Flourishing, which is vital for public policies focused on sustainable human development (Landa-Blanco et al., 2023).

In this sense, exploring predictors of Flourishing can be crucial when working with and selecting the best tools for clinical treatment (VanderWeele et al., 2019). Therefore, the results of this research can serve as a scientific foundation to pave the way for the consideration of implementing practices that promote the expression and development of personal characteristics such as altruism within the creation of programs linked to public policies. By gaining a deeper understanding of the factors involved in the experience of Flourishing states, we contribute to the study of new mechanisms for promoting personal well-being in clinical, social, and everyday contexts (Nugroho et al., 2022; VanderWeele, 2020).

This study provides practical insights for enhancing social well-being and informing policy decisions. Institutions can apply these findings to develop programs that foster Light Triad traits in schools, healthcare centers, and workplaces. For example, schools could integrate curricula that promote emotional intelligence and prosocial behavior, while healthcare centers could emphasize empathy and kindness in patient care. Public policies should prioritize mental health by increasing resources for preventive care and support services while incorporating strength-based approaches that highlight positive traits like empathy and kindness across education, employment, and healthcare systems (Jia et al., 2024; Lines & Jardine, 2025; Müller et al., 2024; Nelson et al., 2024). Targeted interventions aimed at enhancing Flourishing can play a key role in improving psychological well-being by building emotional resilience, strengthening social connections, and fostering a sense of purpose. Given the study’s findings on women’s higher scores in Humanism, future programs could consider tailored approaches that leverage individuals’ strengths to promote leadership and community support, ultimately benefiting both personal and societal well-being.

Despite its theoretical and practical value, this research has several limitations that must be considered. First, the non-probabilistic selection of participants limits the ability to generalize the findings to the broader population. Second, as is common in psychology, the data collection methods rely on self-report measures, which may be influenced by the informants’ introspective abilities and personal biases. Third, the study’s methodological approach did not sufficiently address the biases associated with convenience and snowball sampling. For instance, a key limitation is the relatively young average age of the participants, which was approximately 27 years. This raises questions about whether the results apply to older age groups, as the findings may not generalize beyond this demographic. Fourth, the low reliability of the Kantianism subscale should be considered, as the measure used shows a problematic internal consistency. Fifth, as a cross-sectional study, the data only provide a snapshot at a single time. It is essential to consider longitudinal data to assess whether these patterns hold over time and across different age groups.

Future studies should investigate potential variables mediating the relationship between Light Triad traits and Flourishing, such as self-esteem, social support, physical health, and childhood experiences, among others. Furthermore, considering the subjective nature of Flourishing, future research should adopt mixed-methods approaches that facilitate statistical analysis and offer deeper insights into the personal experiences and interpretations underpinning Flourishing.

## 5. Conclusions

This study indicates that Kantianism, Humanism, and Faith in Humanity are significantly and positively related to levels of Flourishing, which underscore the need for public policies and mental health initiatives that help the development of these traits. Therefore, it is essential to promote incorporating these values and attitudes into the education and development of individuals from an early age (Ellyatt, 2022). This can potentially be achieved through educational programs that promote ethics, empathy, social responsibility, and personal development. However, further studies are necessary to examine these variables more deeply within the Honduran population and establish firm conclusions.

## Figures and Tables

**Table 1:** Comparison of Flourishing and Light Triad Scale scores by gender.

Variable	Group	Mean	*SD*	*t*	*p*	Cohen’s *d*
Flourishing	Men	46.20	8.72	1.15	0.25	0.09
	Women	45.42	8.89			
Faith in Humanity	Men	14.74	3.26	1.62	0.1	0.12
	Women	14.31	3.58			
Humanism	Men	34.81	4.07	−4.77	<0.001	−0.37
	Women	36.17	3.5			
Kantianism	Men	16.15	2.96	−1.07	0.28	−0.08
	Women	16.39	2.86			

*Note:* Degrees of freedom = 714. Based on their effect size, the differences in Flourishing, Faith in Humanity and Kantianism have a very small effect size; the difference in Humanism is classified as small.

**Table 2:** Correlation coefficients between the study variables.

Variable		Flourishing	Faith in Humanity	Humanism	Kantianism
Flourishing	Pearson’s *r.*	—			
	*p*-value	—			
	UB 95%	—			
	LB 95%	—			
Faith in Humanity	Pearson’s *r.*	0.48 ***	—		
	*p*-value	<0.001	—		
	UB 95%	0.53	—		
	LB 95%	0.42	—		
Humanism	Pearson’s *r.*	0.32 ***	0.34 ***	—	
	*p*-value	<0.001	<0.001	—	
	UB 95%	0.38	0.4	—	
	*LB* 95%	0.25	0.27	—	
Kantianism	Pearson’s *r.*	0.33 ***	0.35 ***	0.29 ***	—
	*p*-value	<0.001	<0.001	<0.001	—
	UB 95%	0.39	0.41	0.36	—
	LB 95%	0.26	0.28	0.23	—

*Note*. upper bound = UB, lower bound = LB. *** *p* < 0.001.

**Table 3:** Multiple linear regression model to explain Flourishing scores.

Predictor	Unstandardized *β*	*SE*	Standardized *β*	*t*	*p*
(Intercept)	11.7	2.78	-	4.21	<0.001
Faith in Humanity	0.94	0.09	0.37	10.41	<0.001
Humanism	0.37	0.08	0.16	4.48	<0.001
Kantianism	0.49	0.11	0.16	4.66	<0.001
Gender (Female)	−0.96	0.59	-	−1.63	0.10
Age	−28.6	0.01	−0.06	−1.97	0.05

*Note*: Standardized coefficients can only be calculated for continuous predictors.

## Data Availability

The data supporting the findings of this study are available from the corresponding author upon reasonable request.
